# Exploring Novel Bands and Key Index for Evaluating Leaf Equivalent Water Thickness in Wheat Using Hyperspectra Influenced by Nitrogen

**DOI:** 10.1371/journal.pone.0096352

**Published:** 2014-06-10

**Authors:** Xia Yao, Wenqing Jia, Haiyang Si, Ziqing Guo, Yongchao Tian, Xiaojun Liu, Weixing Cao, Yan Zhu

**Affiliations:** National Engineering and Technology Center for Information Agriculture, Jiangsu Key Laboratory for Information Agriculture, Nanjing Agricultural University, Nanjing, Jiangsu, P. R. China; Oklahoma State University, United States of America

## Abstract

Leaf equivalent water thickness (LEWT) is an important indicator of crop water status. Effectively monitoring the water status of wheat under different nitrogen treatments is important for effective water management in precision agriculture. Trends in the variation of LEWT in wheat plants during plant growth were analyzed based on field experiments in which wheat plants under various water and nitrogen treatments in two consecutive growing seasons. Two-band spectral indices [normalized difference spectral indices (NDSI), ratio spectral indices (RSI), different spectral indices (DSI)], and then three-band spectral indices were established based on the best two-band spectral index within the range of 350–2500 nm to reduce the noise caused by nitrogen and saturation. Then, optimal spectral indices were selected to construct models of LEWT monitoring in wheat. The results showed that the two-band spectral index NDSI(R_1204_, R_1318_) could be used for LEWT monitoring throughout the wheat growth season, but the model performed differently before and after anthesis. Therefore, further two-band spectral indices NDSIb(R_1445_, R_487_), NDSIa(R_1714_, R_1395_)_,_ and NDSI(R_1429_, R_416_), were constructed for the two developmental phases, with NDSI(R_1429_, R_416_) considered to be the best index. Finally, a three-band index (R_1429_−R_416_−R_1865_)/(R_1429_+R_416_+R_1865_), which was superior for monitoring LEWT and reducing the noise caused by nitrogen, was formed on the best two-band spectral index NDSI(R_1429_, R_416_) by adding the 1,865 nm wavelenght as the third band. This produced more uniformity and stable performance compared with the two-band spectral indices in the LEWT model. The results are of technical significance for monitoring the water status of wheat under different nitrogen treatments in precision agriculture.

## Introduction

Real-time, non-destructive monitoring of crop water content based on hyperspectra is an important area of research in precision irrigation in agriculture [1]–[7]. As a widely used measure of crop water status, leaf-equivalent water thickness (LEWT) and canopy-equivalent water thickness (CEWT) not only directly indicate crop water content, but also provide information for leaf area indices. Therefore,they can visually reflect crop water requirements and crop growth status. CEWT has been found to be linearly related to the vegetation water content (VWC), with an R^2^ value of 0.87 for corn [8]. LEWT was shown to have a better linear correlation with reflectance than fuel moisture content (FMC) in the leaves of 10 plant species [9]. In cowpeas, beans, and sugar beet, the R_1300_/R_1450_ leaf water index (ratio of reflectance at 1,300 to 1,450 nm) displayed a characteristic logarithmic correlation with LEWT [10]. During the late period of wheat development (after anthesis), LEWT is more useful than FMC for assessing the water status of wheat. Several optimal water indices for different stages of wheat development are available [11].

Many studies in recent decades have aimed to evaluate the LEWT using remote sensing. Detection of plant water stress through remote sensing has been proposed using indices based on the near-infrared (NIR, 700–1,300 nm) and the middle-infrared (MIR, 1,300–2,500 nm) wavelengths. Hunt et al. found the moisture stress index (MSI) linearly correlated with the log_10_LEWT of *Quercus agrifolia* (sclerophyllous leaves) and *Liquidambar styraciflua* (hardwood deciduous tree leaves), in which the regression equations were different [12]. Ceccato et al. reported that shortwave infrared (SWIR, 1,400–2,500 nm) was sensitive to LEWT, but could not be used alone to determine LEWT because two other leaf parameters (internal structure and dry matter) also influence leaf reflectance in the SWIR [13]. A combination of SWIR and NIR was necessary to determine LEWT. Gao proposed a new vegetation index, the normalized difference water index (NDWI), for the remote sensing of CEWT from space [14]. This index was constructed based on two narrow bands centered near 860 nm and 1,240 nm and was used successfully to detect CEWT and LEWT in cotton and trees [15]–[18]. Based on the difference in reflectance between 945 nm and 975 nm and using Beer's law, Liu et al. calculated the radiation-equivalent water thickness of leaves (RLEWT) [19]. The authors demonstrated that RLEWT was significantly correlated with LEWT. Zarco et al. estimated LEWT from canopy-level reflectance with the simple ratio water index (SRWI), which had a strong correlation with LEWT [20]. Other researchers have proposed the normalized difference infrared index (NDII) [8], [21] and the water index (WI) [22] to estimate LEWT and CEWT.

In addition to these two-band spectral indices, three-band spectral indices have been proposed to evaluate other growth parameters of plants. Schneider et al. [23] and Stow et al. [24] found the visible atmospherically resistant index (VARI) to be minimally sensitive to atmospheric effects and strongly related to live fuel moisture (LFM). Li et al. [25] and Jie et al. [26] found VARI-700 to be significantly correlated with yield at the whole development stages in cotton. Wang et al. constructed three-band vegetation indices, (R_λ1_−R_λ2_+2×R_λ3_)/(R_λ1_−R_λ2_−2×R_λ3_) and (R_λ1_−R_λ2_−R_λ3_)/(R_λ1_+R_λ2_+R_λ3_), to reduce the saturation observed in two-band vegetation indices [27]. They demonstrated that the models for leaf nitrogen content (LNC) using (R_924_−R_703_+2×R_423_)/(R_924_+R_703_−2×R_423_) were stable and accurate and more effective than other published vegetation indices.

Since the value of R_445_ is constant until the total chlorophyll content drops below 0.04 mol m^−2^, Sims et al. added R_445_ to ND_705_ and SR_705_ and developed new three-band spectral indices. The modified ND_705_ and SR_705_ (mND_705_, mSR_705_) were used to predict leaf pigment content, and both were insensitive to species and leaf structure variation [28]. Tian et al. developed a blue nitrogen index (R_434_/(R_496_+R_401_)) and estimated the canopy leaf nitrogen concentration of rice [29].

Water and nitrogen are the main limiting factors in plant growth [30]–[32], and they interact in a complex manner. Previous studies have shown that water and nitrogen are indirectly related (within plants) through chlorophyll and cellulose [33]. It may be effective to construct spectral indices that include a waveband sensitive to chlorophyll or cellulose, which might indirectly eliminate the impact of nitrogen on LEWT monitoring with good performance.

In this study, two experiments were conducted on winter wheat with different water and nitrogen treatments in two consecutive growing seasons. The objectives of the study were: (1) to determine hyperspectral bands that were sensitive to LEWT but insensitive to nitrogen in wheat, (2) to develop new spectral indices for monitoring LEWT in wheat, and (3) to quantify the relationships between LEWT and the new spectral indices to reliably estimate LEWT. These results may provide a technical approach to effectively monitoring of plant water status while minimizing the noise from nitrogen in precision wheat management.

## Materials and Methods

### 2.1 Design of field experiments

Two experiments were conducted at the Pailou Experiment Station at Nanjing Agricultural University, China (118°15′E, 32°1′N). These experiments involved different water (W) and nitrogen (N) treatments. The wheat cultivar Yangmai 18 was grown in two consecutive seasons from November 2010 to June 2011 (a low rainfall season) and from November 2011 to June 2012 (a high rainfall season). Seeds were sown on 1 November 2010 and 3 November 2011 at a density of 180 plants per m^2^ with a plot size of 10 m^2^ (2.5 m×4 m). Each experiment employed a randomized complete block design with three replications. The data from Experiment 1 were used to derive the monitoring models, while the data from Experiment 2 were used to evaluate the models. More details about the W and N treatments, sampling procedures, and environmental conditions are given in Table 1.

**Table 1:** Basic information about different field experiments.

Exp. Number	Year and Variety	Treatment (water treatments (W): %; nitrogen rates (N): kg/hm^2^))	Spectrum and sample data	Soil (yellow brown soil)	Environmental conditions during the growing seasons
Exp. 1 (Calibration data set)	2010–2011 Yangmai 18	**4 W:** W1 (9.5–10.5), W2 (15.5–16.5), W3 (21.5–22.5), W4 (29.5–30.5). **2 N:** N1 (150), N2 (300)	Jointing (3.25), Booting (4.1), Heading (4.12), Anthesis (4.20), Filling (5.6)	Organic matter: 15.5 g kg^−1^, Total N: 1.1 g kg^−1^, Available P: 50.8 mg kg^−1^, Available K: 89.6 mg kg^−1^.	Mean temperature: 10.84°C Maximum temperature:15.75Minimum temperature:5.92Mean Diurnal temperature: 9.83°C Sunshine hours: 1302.5 hPrecipitation:155.14 mm
Exp. 2 (Validation data set)	2011–2012 Yangmai 18	**3 W:** W1 (13.5–14.5), W2 (21.5–22.5), W3 (29.5–30.5). **3 N:** N1 (90), N2 (180), N3 (270)	Jointing (3.24), Booting (4.3), Heading (4.11), Anthesis (4.16), Filling (4.22)	Organic matter: 14.8 g kg^−1^, Total N: 1.1 g kg^−1^, Available P: 50.4 mg kg^−1^, Available K: 88.9 mg kg^−1^.	Mean temperature: 10.98°C Maximum temperature:14.92Minimum temperature:7.04Mean Diurnal temperature: 7.88°C Sunshine hours: 999.003 hPrecipitation:329.51 mm

There were 24 plots in 2010–2011and 27 in 2011–2012. Each plot was constructed from cement with identical length (3 m), width (3 m) and depth (1 m). A transparent plastic sheet was placed over the plots to a height of 3 m above the ground to prevent natural rainfall from flowing into the plot and disturbing the experimental water treatments. From greening until harvest, the runoff from the plastic covers was discharged into a cement drainage channel outside of the plots. From early jointing, the volumetric soil water content was measured with a TRIME-PICO TDR (TRIME-PICO, IMKO, Germany) portable soil moisture speed measuring device in each plot using the five-point method at 4:00 p.m. local time every day. If necessary, water was added immediately to the soil to maintain the designed water content. The date for obtaining spectrum and plant samples was jointing, booting, anthesis, filling.

### 2.2. Data measurements

#### 2.2.1. Measurement of leaf hyperspectral reflectance

Leaf spectral measurements were taken using an accessory of the ASD Field Spec Pro spectrometer (Analytical Spectral Devices, Boulder, CO, USA). The accessory comprises a handheld leaf folder spectral detector with its own light source, which is designed to reduce the effect of time of day, weather, atmospheric vapor pressure, or soil background on the readings. The ASD spectrometer is operated in the 350–2,500 nm spectral region, with a sampling interval of 1.4 nm and spectral resolution of 3 nm between 350 and 1,050 nm, and a sampling interval of 2 nm and spectral resolution of 10 nm between 1,050 and 2,500 nm.

Ten wheat plants were randomly sampled from each plot. The top four leaves on each plant were identified (numbered L1 to L4 starting from the top), and their spectral reflectance was measured. The spectral reflectance averaged over 10 plants at each leaf position was taken as the reflectance for that leaf position. Before the measurement of leaf reflectance in each plot, a standard whiteboard (Labsphere, North Sutton, NH, USA) was used to calibrate the spectral reflectance of the leaves.

#### 2.2.2. Determination of leaf equivalent water thickness

After the leaves were measured for spectral reflectance, they were stored in pre-weighed valve bags in a jar of liquid nitrogen. The leaves were transferred to the laboratory for the determination of leaf area using a portable leaf area meter (LAI-3000, Licor, NE, USA) and their fresh and dry weights were obtained. LEWT was calculated as follows:
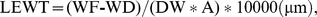
where W_F_ is the leaf fresh weight (g), W_D_ is the leaf dry weight (g), D_W_ is the water density value (g/cm^3^), and A is the leaf area (cm^2^).

#### 2.2.3 Determination of leaf nitrogen content

For each sample, the leaf dry weight was determined by oven-drying the leaves at 80°C to a constant weight. The LNC was determined on a dry weight basis (g 100 g^−1^) using the micro-Kjeldahl method.

### 2.3. Data analysis

The LEWT values and the corresponding leaf spectral reflectance for each sampling date were analyzed with MATLAB 8.2 and Excel 2011 software. The two-band spectral indices considered included the normalized difference spectral index (NDSI, equation (1)), the ratio spectral index (RSI, equation (2)), and the difference spectral index (DSI, equation (3)). To determine the wavelengths in the two-band spectral indices, all combinations of wavelengths within the range of 350–2,500 nm at 1 nm intervals were evaluated according to the criteria presented below:
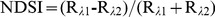
(1)

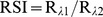
(2)

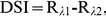
(3)where R_λi_ is spectral reflectance at the wavelength of λi.

For the three-band spectral indices, the best wavelengths were combined with all possible third wavelengths within the range of 350–2,500 nm at 1 nm intervals. The principle of retaining the best wavebands of the three-band indices has been applied in previous studies [26]–[27]. Three classical three-band spectral indices based on the NDSI were considered and constructed (equations (4) to (6)).
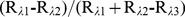
(4)

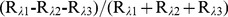
(5)

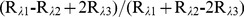
(6)


The criteria for evaluating the best wavelengths were the coefficient of determination (R^2^), standard error (SE), and relative root mean square error (RRMSE) [25].

The RRMSE was calculated as follows:
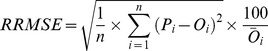
(7)where *P_i_* and *O_i_* are the predicted and observed values, respectively, 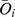 is the observed mean value, and *n* is the number of samples.

## Results

### 3.1 Variation in LEWT

**Figure 1:**
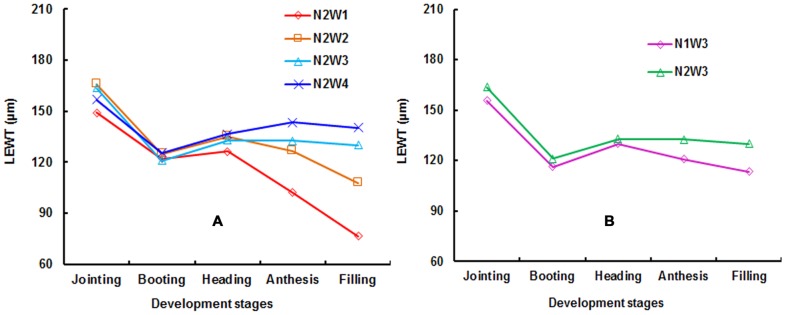
Changes of LEWT under different water treatments at N2 (A) and varied nitrogen treatments at W3 (B) in Exp. 1.

Fig. 1A shows how LEWT varied under the same nitrogen level and different water treatments in Exp. 1. There was a greater difference at anthesis and filling than at booting, jointing, or heading due to water treatment, with LEWT values in the order of W4 > W3 > W2 > W1, which is normal for irrigation scenarios. During the late stages of plant development, evaporation increased with the air temperature, which resulted in greater differences in LEWT among water treatments. Drought stress (W1, W2) was found to accelerate plant development, and LEWT value was higher than in W3 (normal amounts of water), while the W4 treatment was found to enhance the acceleration of plant development.

Fig. 1B displays LEWT for the W3 treatment for leaf L1 at various nitrogen levels. LEWT was greater at N2 than at N1, which was also the case for the W1, W2, and W4 treatments. Due to the synergistic effects of water and nitrogen on the growth processes of wheat, high-level nitrogen treatment promoted water uptake, resulting in a greater LEWT compared to low nitrogen levels [32]–[33]. Similar results were obtained for L2, L3, and L4 in Exp. 2.

### 3.2 Variations in leaf hyperspectral reflectance

**Figure 2:**
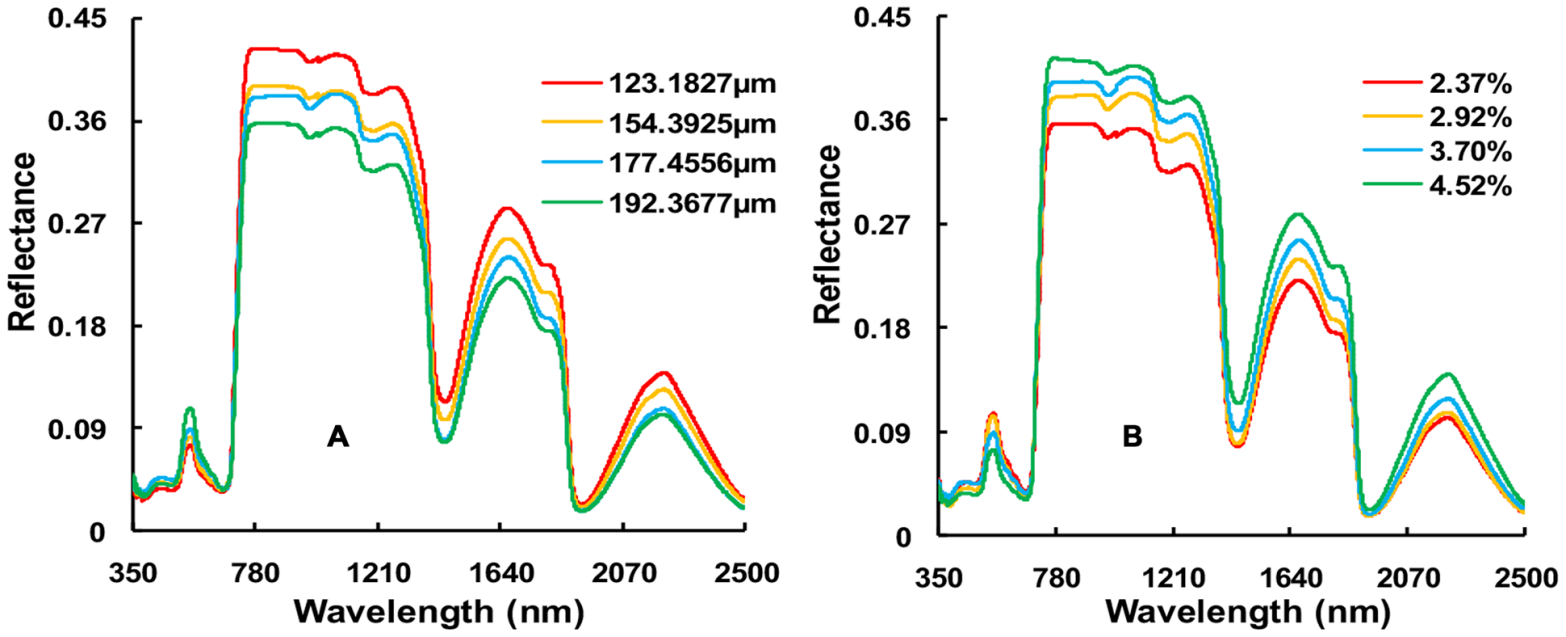
Reflectance of leaves under different LEWT at N2 (A) and LNC at W4 (B) in Exp. 1.

Differences in LEWT and LNC values significantly affected the leaf hyperspectral reflectance of wheat. Fig. 2A shows leaf reflectance at different LEWT levels for N2 during the booting stage in Exp. 1. Leaf reflectance in the visible range was not significantly affected by LEWT. However, in the NIR range, especially at the central wavelengths of 900 nm, 1,200 nm, 1,400 nm, 1,450 nm, and 1,930 nm, leaf reflectance significantly decreased as LEWT increased. This result suggests that nitrogen levels influenced the variations in leaf hyperspectral reflectance. Thus the reflectance spectrum used for monitoring crop water may contain some information due to the presence of nitrogen, which should be eliminated or reduced.

Fig. 2B shows the reflectance at various LNC levels for the W4 treatment. N levels and W treatments markedly influenced the characteristics of spectral reflectance, with different spectral responses in the various waveband regions. With increasing N levels, the reflectance decreased in the visible bands and increased in the near infrared, with obvious differences among the four N levels. This result implies that these spectral regions are relatively sensitive to the growth status of wheat at different N levels. In the visible range, differences in reflectance appeared mainly at wavelengths of 450 nm and 700 nm, the central wavelengths of pigment absorption. The reflectance increased as LNC increased in the near infrared, particularly at 970 nm and 1,200 nm, the central wavelengths of water absorption.

### 3.3 Correlation of LEWT and LNC with the original and first derivative hyperspectra in wheat leaves

**Figure 3:**
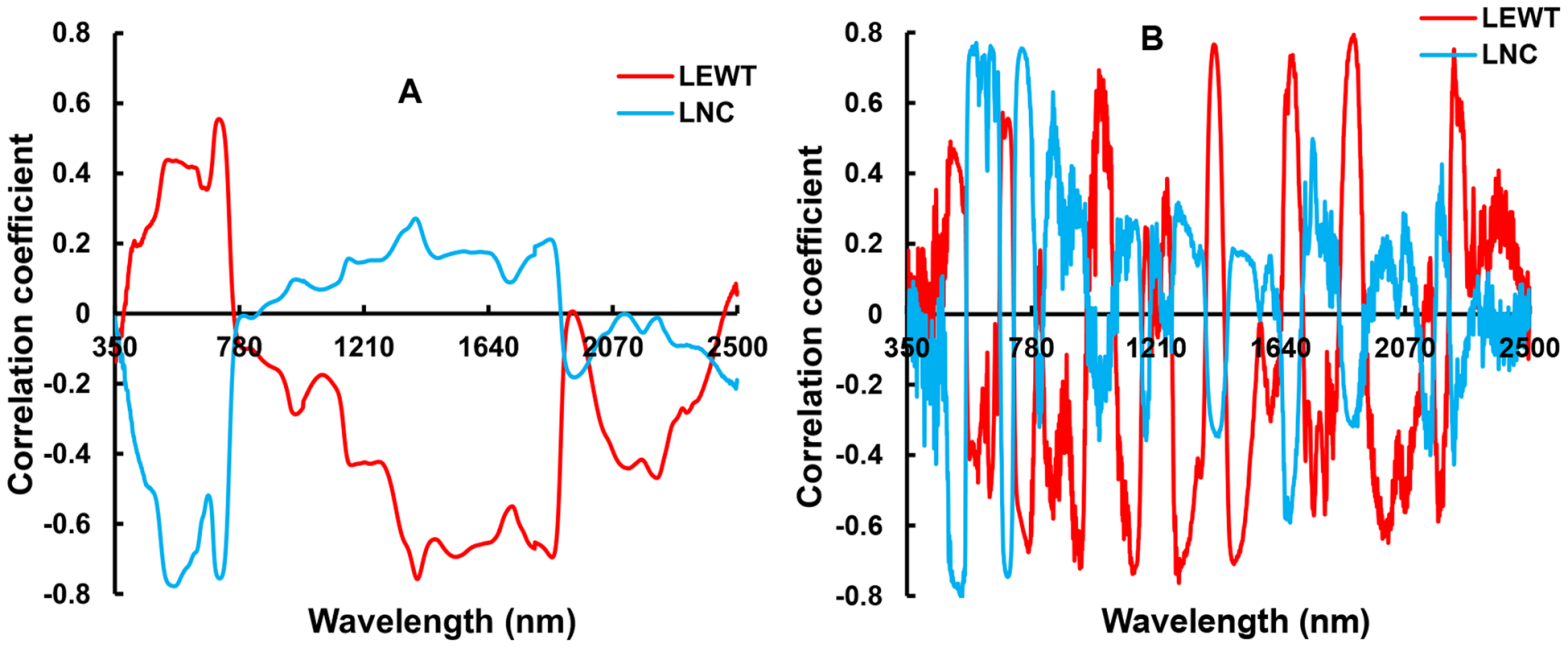
The correlation of LEWT and LNC to original spectral reflectance (A) and its first derivative (B) in Exp. 1.

The correlation of LEWT and LNC with the original spectrum and its first derivative spectrum was determined based on the observed data from Exp.1. Fig. 3 shows that LEWT and the original spectral reflectance were positively correlated in the visible region and negatively correlated in the NIR, with peaks of the absolute value of the correlation at 970 nm, 1,200 nm, 1,395 nm, 1,450 nm, 1,850 nm, and 2,200 nm. The strongest correlation was at 1,395 nm (R_1395_ = −0.7576). This result indicates that LEWT markedly influenced the reflectance of wheat leaves, with different spectral responses in the various waveband regions. In general, the correlation between LNC and the original spectral reflectance generally had the opposite sign to the correlation of LEWT.

Fig. 3A shows that the largest 10% of |R|_LEWT_ and |R|_LNC_ overlapped at 1,365–1,424 nm and at 1,839–1,869 nm in the NIR region. The largest 20% of |R_LEWT_| and |R_LNC_| overlapped in two regions of the visible bands, 526–566 nm and 701–723 nm (Fig. 3A). These results indicate that the wavelengths sensitive to both LEWT and LNC were mainly in the visible and NIR range. However, the largest 20% of |R_LEWT_| did not overlap anywhere, with the smallest values of |R_LNC_|, suggesting that no wavelength was sensitive to LEWT and insensitive to LNC in the same region.

The first derivatives of the original spectrum and LEWT were also strongly correlated in the NIR range, especially at the central wavelengths of 1,890 nm, 1,300 nm, and 1,400 nm (Fig. 3B). The largest 10% of |R_LEWT_| overlapped with |R_LNC_| at 770 nm (Fig. 3B). In the original spectrum, there was no overlap between the largest 20% of |R_LEWT_| and the smallest values of |R_LNC_|. No wavelength in the first derivative was sensitive to LEWT and insensitive to LNC simutaneously.

### 3.4 The relationships of spectral indices to LEWT

#### 3.4.1. Relating two-band spectral indices to LEWT during the entire growth period

**Figure 4:**
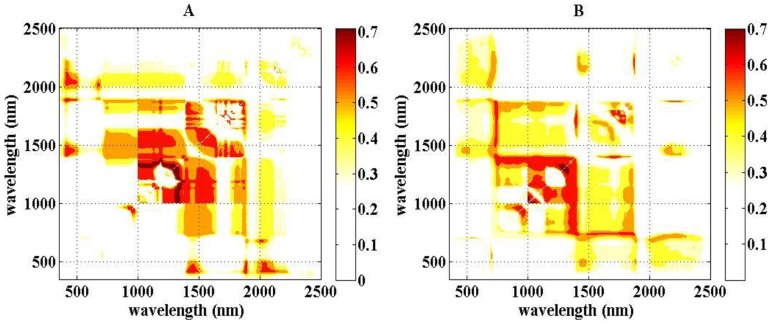
Contour map of coefficients of determination (R^2^) for linear relationship between NDSI and LEWT (A: calibration dataset, B: validation dataset).

**Figure 5:**
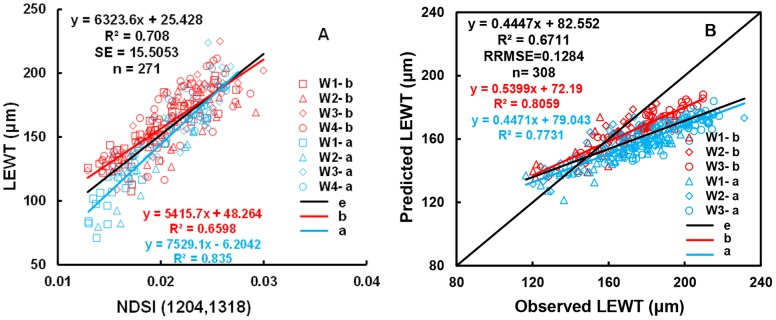
Quantitative relationships between NDSI (R_1204_, R_1318_) and LEWT (A); the 1∶1 relationship between the predicted and observed LEWT based on NDSI (R_1204_, R_1318_) (B).

Contour maps of R^2^ values for the relationship of LEWT during the full growth period with the two wavelengths in the two-band indices were constructed (not shown). The results for NDSI and RSI were similar, while R^2^ values were lower for DSI. The top 10% of R^2^ values for NDSI, using the calibration data, were mainly in the ranges of λ1 = 1,000–1,320 nm and λ2 = 1,300–1,350 nm (Fig. 4A). Using the validation data, the top 10% of R^2^ values were in the ranges of λ1 = 1,205–1,315 nm and λ2 = 1,287–1,337 nm (Fig. 4B). Finally, NDSI (R_1204_, R_1318_) was selected based on the overlapping region. This index had an R^2^ value of 0.708 and an SE of 15.5053 µm (Fig. 5A). Fig. 5B shows the relationship between the predicted and observed LEWT. There are clear differences in the fit to the data before and after anthesis, which may be due to metabolism (especially the nitrogen content) and changes in position of the plants.

Further statistical analysis was conducted to determine whether the two linear relationships could be grouped. Thereby, a linear parallel curve analysis with grouped data was used to determine whether the critical curves for LEWT differed before and after anthesis [34]. The results showed that the F value for the LEWT before and after anthesis was significantly different from that of the residual. The F value for NDSI(R_1204_, R_1318_)×LEWT before anthesis and NDSI(R_1204_, R_1318_)×LEWT after anthesis also differed significantly from that of the residual (Table 2).

**Table 2:** Simple grouping linear analysis of the model between NDSI and LEWT in wheat before and after anthesis.

Developmental stage			NDSI (R_1204_,R_1318_)×developmental stage			Residual		
df	MS	F	df	MS	F	df	MS	F
1	7478.7	37.34*	1	4202.6	20.98*	267	200.3	6.76

**Notes:**

**df:** degrees of freedom.

**MS:** mean square.

*: ***P***<0.05.

#### 3.4.2. Relating NDSI (R_ë1_, R_ë2_) to LEWT before and after anthesis

**Figure 6:**
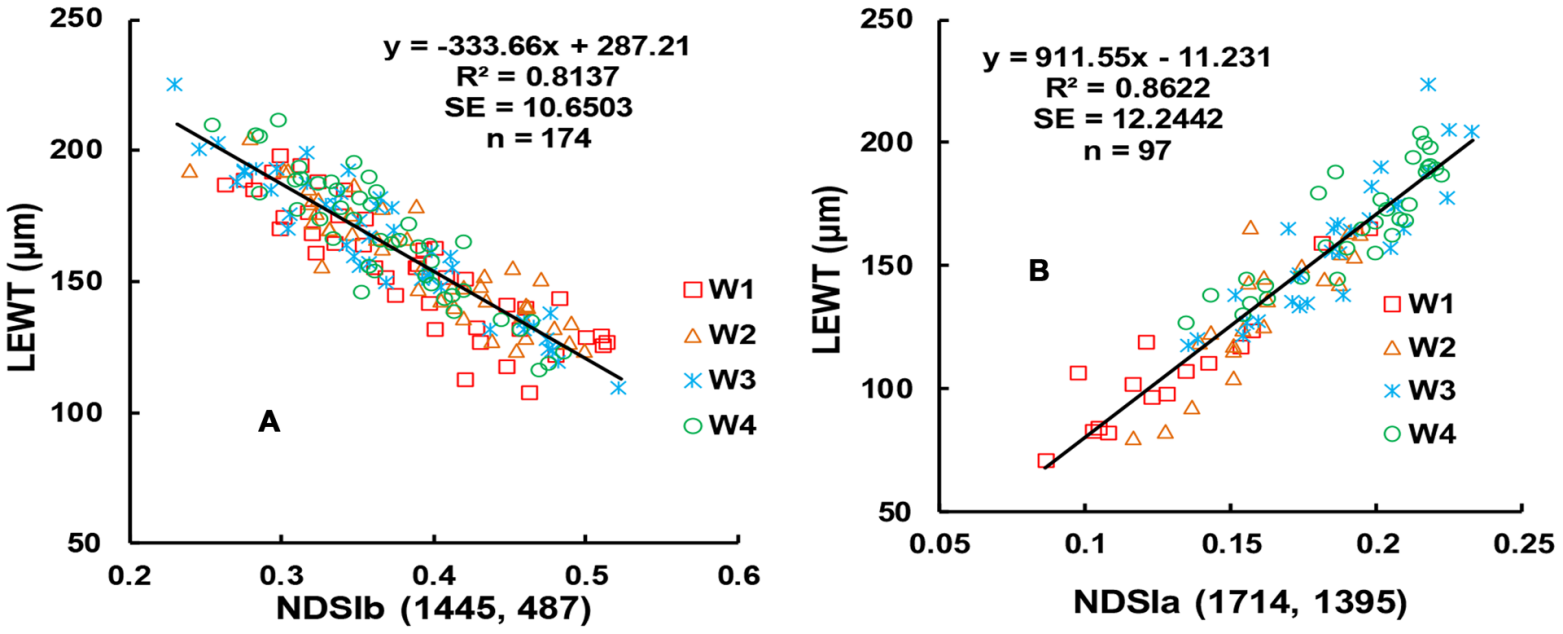
Contour map of the linear relationship with top 15% R^2^ between NDSI and LEWT before (A) and after (B) anthesis, and the common area between A and B (C).

**Figure 7:**
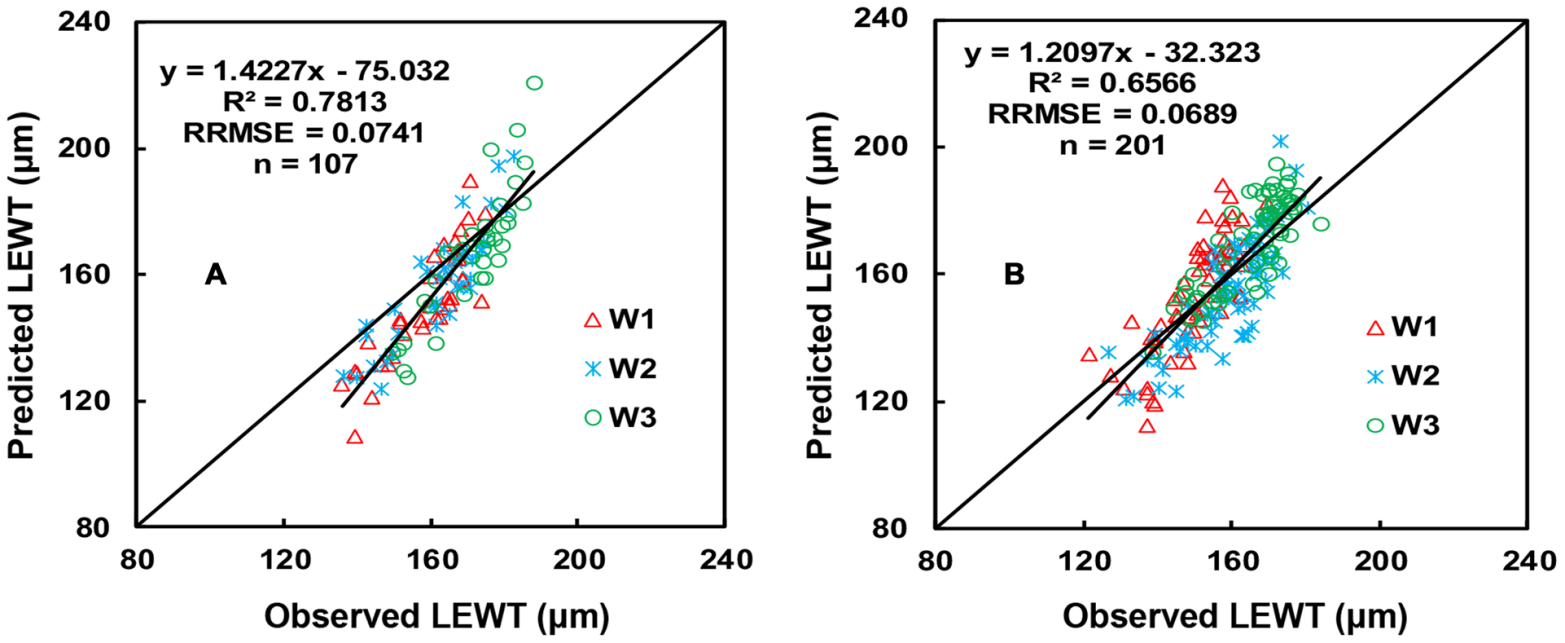
Quantitative relationships between NDSIe (R_1445_, R_487_) and LEWT before anthesis (A); quantitative relationships between NDSIt (R_1714_, R_1395_) to LEWT after anthesis (B).

Based on the above results, the developmental stages were considered separately in further studies of the relationship between LEWT and the NDSI (R_ë1_, R_ë2_). The optimal wavelengths for the period before anthesis were 1,445 and 487, giving the spectral index NDSIb(R_1445_, R_487_) (NDSI before anthesis). This index had an R^2^ value of 0.8137 and an SE of 10.6503 µm. The optimal wavelengths for the period after anthesis were 1,714 and 1,395, giving the spectral index NDSIa(R_1714_, R_1395_) (NDSI after anthesis). For this index, the R^2^ value was 0.8622 and the SE was 12.2442 µm (Fig. 6). The independent dataset from Exp. 2 was used to test the models for LEWT before and after anthesis (Fig. 7). The results showed that NDSIb and NDSIa predicted LEWT more accurately than did the spectral index NDSI (R_1204_, R_1318_) for the entire growth period and the spectral index NDSI(R_1429_, R_416_) of the common sensitive areas. In the optimal spectral indices NDSIb(R_1445_, R_487_) and NDSIa(R_1714_, R_1395_), 1,445 nm and 1,395 nm were the most sensitive wavelengths to LEWT, as explained above. The bands at 487 nm and 1,714 nm were sensitive to chlorophyll and lignin.

**Figure 8:**
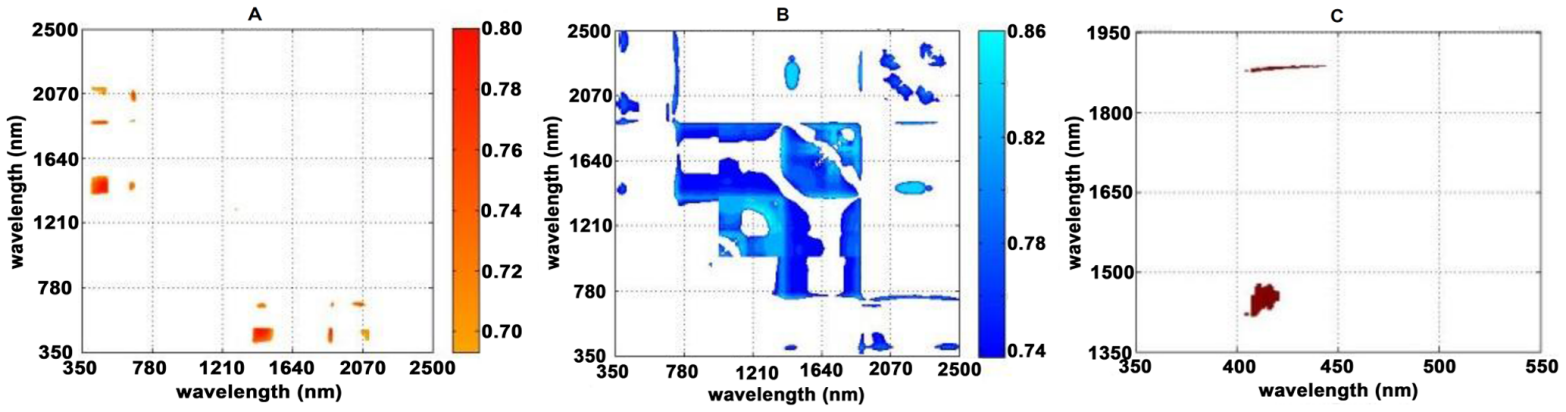
The 1∶1 relationship between the predicted and observed LEWT based on NDSIb (R_1445_, R_487_) for wheat before anthesis (A); the 1∶1 relationship between the predicted and observed LEWT based on NDSIa (R_1714_, R_1395_) for wheat after anthesis (B).

**Figure 9:**
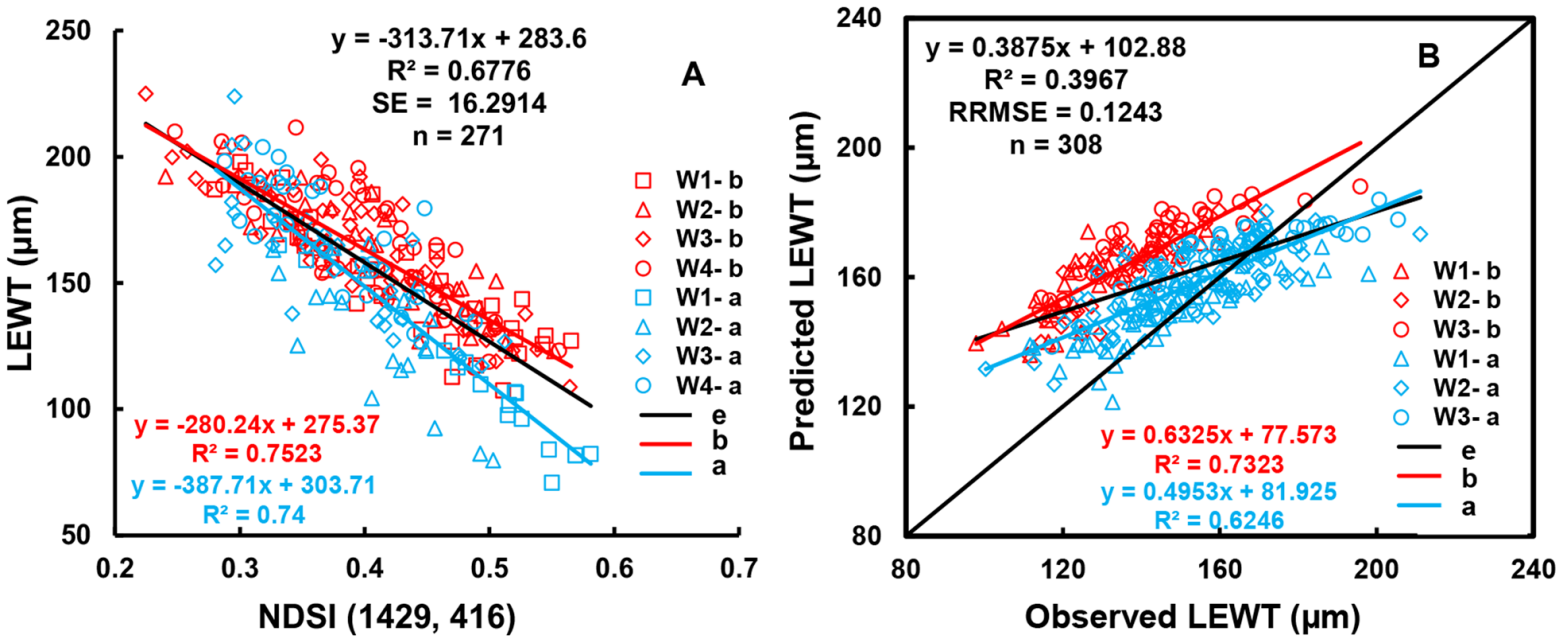
Quantitative relationships between (R_1429_−R_416_)/(R_1429_+R_416_) and LEWT (A); the 1∶1 relationship between the predicted and observed LEWT on (R_1429_−R_416_)/(R_1429_+R_416_) in wheat (B).

The indices NDSIb(R_1445_, R_487_) and NDSIa(R_1714_, R_1395_) contain four wavelengths, which will increase the cost of manufacturing-self. This motivated us to search for common two-band wavelengths before and after anthesis. The central waveband ranges of NDSI before and after anthesis differed significantly, and the top 10% of the optimal values of R^2^ had no common area (Figures were omitted). However, the top 15% of R^2^ values before (Fig. 8A) and after (Fig. 8B) anthesis had two common areas, with the first λ1 in the range of 405–420 nm and λ_2_ in the range of 1,418–1,477 nm and the second λ1 in the range of 408–443 nm and λ_2_ in the range of 1,876–1,886 nm (Fig. 8C). Based on the R^2^, SE, and RRMSE of the calibration and validation data, the spectral index NDSI(R_1429_, R_416_) was selected for the periods both before and after anthesis. The calibration of LEWT both before and after anthesis had an R^2^ value of 0.6776 and an SE of 16.2914, and the prediction using the validation data had an R^2^ value of 0.3967 and an RRMSE of 0.1243 (Fig. 9, Table 3).

**Table 3:** Relationships of spectral indices to LEWT (n = 271) and testing performances (n = 308) in wheat.

Spectral index		Growth stage	Calibration(n = 271)		Validation to LEWT (n = 308)		Validation to LNC (n = 308)	
			R^2^	SE	R^2^	RRMSE	R^2^	RRMSE
2-band	NDSI (R_1204_, R_1318_)	e	0.7080	15.5053	0.6711	0.1284	0.1451	42.6089
	NDSIb (R_1445_, R_487_)	b	0.8137	10.6503	0.7813	0.0741	0.2221	36.0804
	NDSIa (R_1395_, R_1714_)	a	0.8622	12.2442	0.6566	0.0689	0.2272	40.0445
	NDSI (R_1429_,R _416_)	e	0.6776	16.2914	0.3967	0.1243	0.2075	35.9918
**3-band**	**(R_1429_−R_416_−R_1865_)/(R_1429_+R_416_+R_1865_)**	**e**	**0.6905**	**15.9613**	**0.7255**	**0.0689**	**0.0849**	**39.7950**
	(R_1429_−R_416_)/(R_1429_+R_416_−R_1883_)	e	0.7093	15.4695	0.3860	0.0994	0.1632	36.9978
	(R_1429_−R_416_+2×R_1435_)/(R_1429_+R_416_−2×R_1435_)	e	0.6260	17.5466	0.3375	0.1117	0.1858	36.0329
Previous	WI (R_900_, R_970_) [22]	e	0.5260	19.7528	0.6480	0.2241	0.1518	47.2203
	SRWI (R_858_, R_1240_) [20]	e	0.1625	26.2569	0.4932	0.0614	0.1315	39.7522
	MSI (R_1600_, R_820_) [12], [13]	e	0.4982	20.3248	0.5206	0.2915	0.0599	50.0599
	NDWI (R_860_, R_1240_) [14]	e	0.1604	26.2907	0.4954	0.0613	0.1295	39.7553
	NDII (R_850_, R_1650_) [8], [21]	e	0.2388	25.0332	0.5104	0.2953	0.0562	50.1623

**Notes:**

b represents before anthesis,

a represents after anthesis,

e represents the entire growth period.

#### 3.4.3. Relating three-band spectral indices based on NDSI(R_1429_, R_416_) to LEWT

**Figure 10:**
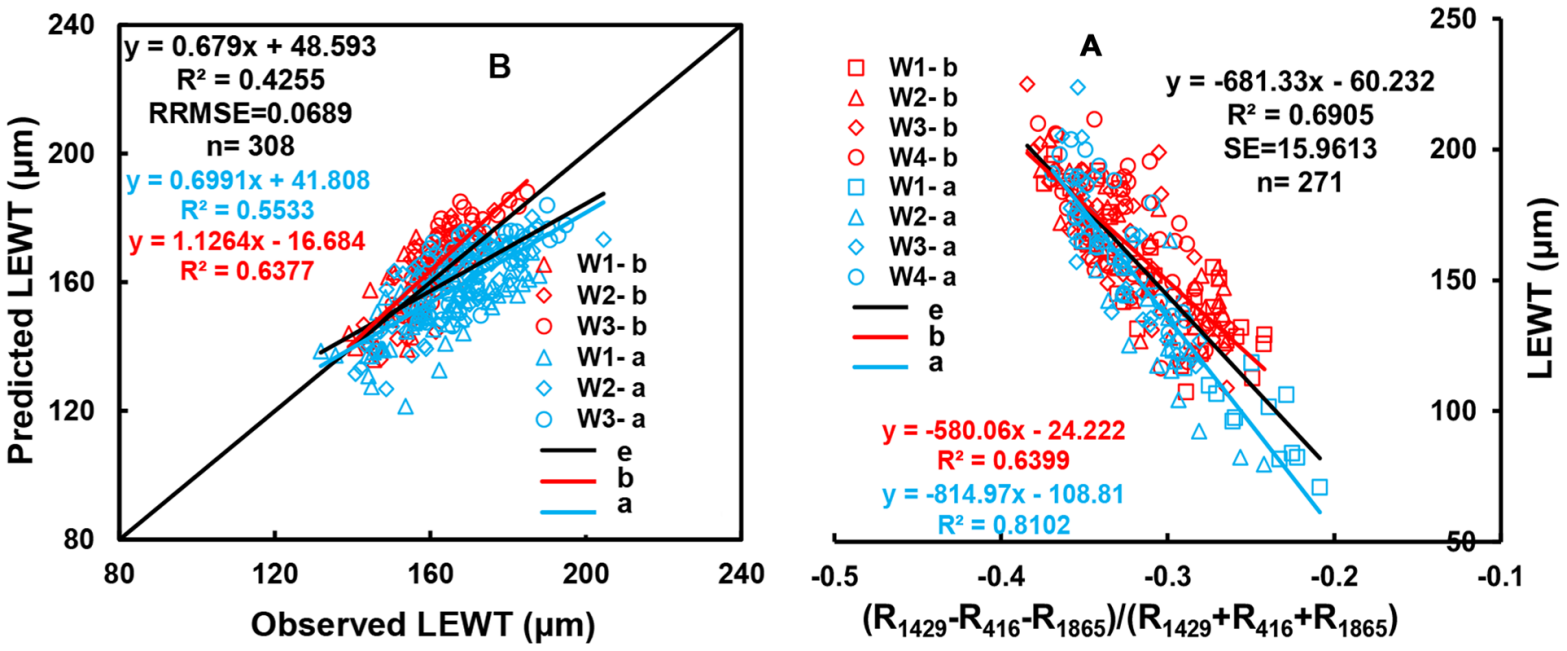
Quantitative relationships between (R_1429_−R_416_−R_1865_)/(R_1429_+R_416_+R_1865_) between LEWT in wheat (A); the 1∶1 relationship between the predicted and observed LEWT on (R_1429_−R_416_−R_1865_)/(R_1429_+R_416_+R_1865_) (B).

The third band (λ3) can help to reduce both the effect of nitrogen and the effect of saturation. The best three-band index, using the best wavelengths for NDSI, was (R_1429_−R_416_−R_1865_)/(R_1429_+R_416_+R_1865_). The third band (at 1,865 nm) located in the SWIR is close to the sensitive band of cellulose (1,880 nm) (Table 3). However, the performance of the model for the three-band index for NDSIb(R_1445_, R_487_) and NDSIa(R_1714_, R_1395_). The cost of developing the equipment in the future will decrease as its use on a large scale becomes more feasible due to the need for fewer wavelengths. Moreover, the three-band index model displayed a marked improvement compared with the model based on NDSI(R_1429_, R_416_) (Fig. 9, 10).

#### 3.4.4. Comparison of the performance of the new and previous models

Five popular existing spectral indices that are used to predict crop water content were compared with the spectral indices developed in the current study. As shown in Table 3, of the five indices, WI, MSI, and NDII yielded the best predictions of LEWT, and WI performed especially well. However, the two spectral indices developed in this study, NDSIb and NDSIa, performed considerably better than all of the existing indices. This result may be due to the fact that the existing spectral indices are based on the relative water content of leaves and whole plants of various crops, whereas the spectral indices developed in the current study were constructed based on the LEWT in wheat.

## Discussion

### 4.1. Response of leaf spectral reflectance to water and nitrogen treatments in wheat

Previous studies of the response of leaf spectra to different water conditions and nitrogen levels have shown that the sensitivity of leaf spectral reflectance to water content peaks at the central wavelengths of 970 nm, 1,200 nm, 1,400 nm, 1,450 nm, 1,730 nm, 1,930 nm, 2,100 nm, and 2,500 nm [35]. Within the range of 1,300–2,500 nm, spectral reflectance is affected by water in the plants, which directly absorbs radiation. Within the range of 400–1,300 nm, reflectance is influenced by changes in the internal structure of the leaf, which is a result of their different water content ratios [7], [36]. The influence of nitrogen on spectral reflectance occurs mainly in the range of the visible and NIR wavebands; and the wavebands most sensitive to nitrogen are in the range of 530–560 nm [37]–[38]. In the present study, leaf hyperspectral reflectance at different LEWT levels in the N2 treatment did not change significantly in the visible band range. However, reflectance significantly decreased as LEWT increased within the NIR range, especially at 900 nm, 1,200 nm, 1,400 nm, 1,450 nm, 1,730 nm, and 1,930 nm. Leaf hyperspectral reflectance increased with increasing LNC within the NIR range, and it decreased considerably at the central wavelengths of water absorption. Therefore, to monitor plant water content, some sensitive wavelength and spectral indices that are strongly correlated with LEWT, but less well correlated with LNC, should be extracted with consideration of the fact that the spectrum reflectance is affected by both water and nitrogen.

### 4.2. Two-band spectral indices sensitive to LEWT in wheat

The systematic analysis of two-band spectral indices sensitive to LEWT was conducted for the entire growth. NDSI is more closely related to LEWT than RSI or DSI. NDSI(R_1204_, R_1318_) was the optimal spectral index for predicting LEWT in wheat during the whole growth period. As earlier analyses have indicated, differences in LNC before and after anthesis might have an impact on the accuracy of the LEWT model throughout plant growth. Therefore, separate indices were proposed for the periods before and after anthesis, namely, NDSIb(R_1445_, R_487_) and NDSIa(R_1714_, R_1395_), respectively. The wavelengths 1,445 nm and 1,395 nm were the most sensitive to LEWT, while the wavelengths 487 nm and 1,714 nm were sensitive to chlorophyll and lignin, respectively. This indicated that LEWT models before and after anthesis were established separately, while the impact of nitrogen was eliminated by the use of wavebands that were sensitive to chlorophyll or lignin, as reported previous [33].

### 4.3. Three-band spectral indices sensitive to LEWT in wheat

Three-band indices contain less noise due to chlorophyll or lignin than two-band spectral indices [23]–[24], [28]. They also display less saturation than two-band spectral indices [27]. Therefore, to reduce the noise caused by nitrogen before and after anthesis in the LEWT model, the three-band spectral index (R_1429_−R_416_−R_1865_)/(R_1429_+R_416_+R_1865_) was developed based on the best overall two-band spectral index NDSI(R_1429_, R_416_). Although the model based on the three-band index was a poorer predictor than those based on NDSI (R_1204_, R_1318_) or on NDSIb(R_1445_, R_487_) and NDSIa(R_1714_, R_1395_), the calibration and validation on the resulting scatter plot was centralized, and the LEWT model was more uniform and stable with a lower RRMSE. The results also showed that the three-band spectral indices (R_1429_−R_416_−R_1865_)/(R_1429_+R_416_+R_1865_) clearly reduced the noise due to nitrogen in the LEWT model throughout the whole period of wheat growth, with an R^2^ value of 0.7255and RRMSE of 0.0689 for LEWT validation, and an R^2^ value of 0.0849 and an RRMSE of 39.7950 for LNC validation.

## Conclusions

Previous studies have shown that the transportation of amino acids from leaves to grains after anthesis leads to physiological and biochemical changes in the organizational structures of leaves, which affect LEWT monitoring based on leaf hyperspectral reflectance. In this study, we demonstrated that different water and nitrogen treatments affected the variation in LEWT and the leaf hyperspectral reflectance of wheat within the 350–2,500 nm range. Furthermore, the top 10% of the maximum |R_LEWT_| and |R_LNC_| values were found to share common wavelength ranges. Based on this study and previous reports, when monitoring LEWT, the noise from the LNC should be considered. The model based on a three-band index (R_1429_−R_416_−R_1865_)/(R_1429_+R_416_+R_1865_) described in this paper performed well for monitoring LEWT, with a higher predictability and stability for water content and lower noise levels due to nitrogen under various water and nitrogen treatments.
